# Dicalcium silicate-induced mitochondrial dysfunction and autophagy-mediated macrophagic inflammation promotes osteogenic differentiation of BMSCs

**DOI:** 10.1093/rb/rbab075

**Published:** 2021-12-13

**Authors:** Qianting Luo, Xingyang Li, Wenchao Zhong, Wei Cao, Mingjing Zhu, Antong Wu, Wanyi Chen, Zhitong Ye, Qiao Han, Duraipandy Natarajan, Janak L Pathak, Qingbin Zhang

**Affiliations:** 1 Department of Temporomandibular Joint, Affiliated Stomatology Hospital of Guangzhou Medical University, Guangdong Engineering Research Center of Oral Restoration and Reconstruction, Guangzhou Key Laboratory of Basic and Applied Research of Oral Regenerative Medicine, Guangzhou 510182, China; 2 Jiangmen Central Hospital, Affiliated Jiangmen Hospital of Sun Yat-sen University, Jiangmen 529030, China; 3 Department of Oral Cell Biology, Academic Centre of Dentistry Amsterdam (ACTA), Vrije Universiteit Amsterdam and University of Amsterdam, Amsterdam 1081LA, The Netherlands; 4 Affiliated Stomatology Hospital of Guangzhou Medical University, Guangdong Engineering Research Center of Oral Restoration and Reconstruction, Guangzhou Key Laboratory of Basic and Applied Research of Oral Regenerative Medicine, Guangzhou 510182, China

**Keywords:** dicalcium silicate, macrophagic inflammation, mitochondrial function, autophagy, osteogenesis

## Abstract

Dicalcium silicate (Ca_2_SiO_4_, C_2_S) has osteogenic potential but induces macrophagic inflammation. Mitochondrial function plays a vital role in macrophage polarization and macrophagic inflammation. The mitochondrial function of C_2_S-treated macrophages is still unclear. This study hypothesized: (i) the C_2_S modulates mitochondrial function and autophagy in macrophages to regulate macrophagic inflammation, and (ii) C_2_S-induced macrophagic inflammation regulates osteogenesis. We used RAW264.7 cells as a model of macrophage. The C_2_S (75–150 μg/ml) extract was used to analyze the macrophagic mitochondrial function and macrophage-mediated effect on osteogenic differentiation of mouse bone marrow-derived mesenchymal stem cells (BMSCs). The results showed that C_2_S extract (150 μg/ml) induced TNF-α, IL-1β and IL-6 production in macrophages. C_2_S extract (150 μg/ml) enhanced reactive oxygen species level and intracellular calcium level but reduced mitochondrial membrane potential and ATP production. TEM images showed reduced mitochondrial abundance and altered the mitochondrial morphology in C_2_S (150 μg/ml)-treated macrophages. Protein level expression of PINK1, Parkin, Beclin1 and LC3 was upregulated but TOMM20 was downregulated. mRNA sequencing and KEGG analysis showed that C_2_S-induced differentially expressed mRNAs in macrophages were mainly distributed in the essential signaling pathways involved in mitochondrial function and autophagy. The conditioned medium from C_2_S-treated macrophage robustly promoted osteogenic differentiation in BMSCs. In conclusion, our results indicate mitochondrial dysfunction and autophagy as the possible mechanism of C_2_S-induced macrophagic inflammation. The promotion of osteogenic differentiation of BMSCs by the C_2_S-induced macrophagic inflammation suggests the potential application of C_2_S in developing immunomodulatory bone grafts.

## Introduction

Dicalcium silicate (Ca_2_SiO_4_, C_2_S) is widely used for coating orthopedic and dental implants [1]. C_2_S is a biomaterial with good biocompatibility and no obvious cytotoxicity to RAW264.7, THP-1, MC3T3-E1 and other cells [2–5]. C_2_S is used as dental cavity capping material and drug carrier [1, 6]. C_2_S induces osteoblast differentiation and inhibits osteoclastogenesis [6, 7]. Inorganic materials of the grafted biomaterials induce host immune reaction that affects immune cells’ function-mediated immunomodulation [8]. Only a few studies are focused on C_2_S-mediated host immune responses [9]. Shreds of literature had reported the immunomodulatory property of C_2_S mainly via affecting macrophage polarization and functions [2, 4, 10–12]. The exposure of biomaterial regulates macrophage polarization [11, 13]. M1 macrophages are inflammatory macrophages that release proinflammatory cytokines IL-1β, IL-6, TNF-α, etc. On the contrary, M2 macrophages are anti-inflammatory macrophages that release anti-inflammatory cytokines IL-4, IL-10, TGF-β, etc [14, 15]. Various signaling pathways including, mitochondrial metabolism and cell metabolism are involved in macrophage polarization [15, 16]. Many studies including ours revealed that C_2_S triggers macrophagic inflammation and the release of inflammatory cytokines [2, 10]. However, the molecular mechanism of C_2_S-induced macrophagic inflammation is still unclear.

Mounting evidence indicates that mitochondrial functions are involved in macrophage functions and immune responses [17–19]. Mitochondrial dysfunction had been reported to prevent repolarization of polarized macrophages [16]. However, the mitochondrial function in C_2_S-induced macrophagic inflammation is still unclear. Therefore, it is important to investigate the mitochondrial function of C_2_S-treated macrophages and the role of macrophagic inflammation on bone regeneration.

During the initial stage of bone defect healing, acute inflammation is essential to trigger bone regeneration [20–22]. A low level of macrophagic inflammation promotes osteogenesis via autophagy regulation [23]. Lack of recruitment and activation of macrophages impairs bone defect healing in animal models indicating the significant role of macrophages in bone regeneration [24]. However, prolonged macrophagic inflammation impedes bone repair [25, 26]. This might be related to chronic inflammation caused by the failure of switching of macrophages from M1 to M2 phenotype [16] and the failure in the spatiotemporal expression of proinflammatory cytokines [27–31]. There is an opposing role of proinflammatory cytokines such as interleukin (IL)-1β, IL-6 and tumor necrosis factor-alpha (TNF-α) that could inhibit osteogenic differentiation and some findings reported that these cytokines could promote osteogenic differentiation [25–27]. Amorphous calcium phosphate nanoparticles-induced macrophagic inflammation had been recently reported to weaken osteogenic differentiation bone marrow-derived mesenchymal stem cells (BMSCs) [32, 33]. Our previous research results showed that C_2_S induces inflammation in RAW264.7 cells and promotes the release of TNF-α, IL-1β and IL-6 through toll-like receptor-2 (TLR2)-mediated nuclear factor kappa-light-chain-enhancer of activated B cells (NF-κB) and c-Jun N-terminal kinase (JNK) pathways [2]. Horeover, the effect of C_2_S-induced macrophagic inflammation on osteogenic differentiation of precursor cells is still unknown.

Our previous studies focused on investigating the osteogenic potential of C_2_S and the underlying mechanisms [7]. In this study, we aimed to elucidate the role of mitochondrial function in C_2_S-induced macrophagic inflammation as well as the role of C_2_S-induced macrophagic inflammation on the osteogenic differentiation of precursor cells. Our findings revealed that C_2_S regulates macrophagic inflammation via inducing mitochondrial dysfunction and autophagy. Interestingly, C_2_S-induced macrophagic inflammation robustly promoted osteogenic differentiation of precursor cells.

## Materials and methods

### Characterization of C_2_S and C_2_S extract preparation

C_2_S is a well-characterized biomaterial frequently used in dental capping and drug delivery [1, 6]. Our previous studies had performed characterization and analysis of the C_2_S micro-particles used in this experiment [2, 7, 10]. C_2_S micro-particles were synthesized and characterized in the Shanghai Institute of Ceramics, Chinese Academy of Sciences as described previously [7]. C_2_S particles with a purity of 99% were obtained. Different concentrations (75, 100, 125, or 150 μg/ml) of C_2_S particles were dispersed in high glucose Dulbecco’s modified eagle medium (DMEM) and sonicated for 4 h to prepare the C_2_S extracts [7]. The C_2_S extracts were filtered with a 0.22-μm filter and stored at 4°C.

### Macrophage cell culture

The mouse macrophage cell line, RAW264.7 (National Collection of Authenticated Cell Cultures, TCM13, Shanghai, China) was used as a model of macrophage. The cells were maintained in high glucose DMEM (Gibco, USA) supplemented with 10% fetal bovine serum (Gibco, USA) and 1% penicillin-streptomycin solution (Gibco, USA), under 37°C, supplied with 5% CO_2_ and 95% air atmosphere.

### Effect of C_2_S extract on macrophage vitality

Macrophages (3000 cells/well) were cultured in 96-well plates in presence of 0, 75, 100, 125 or 150 μg/ml C_2_S extract groups and incubated the cells for 4, 24, 48 and 72 h. The cell vitality was evaluated using the cell counting kit-8 assay (CCK8, BestBio, China) as described previously [7].

### Cytokine expression analysis by ELISA

After 24 h culture of macrophages with C_2_S extract (150 μg/ml), the conditioned medium was collected and centrifuged at 3000 rpm for 10 min. Levels of TNF-α, IL-1β and IL-6 were measured in the supernatant using mouse ELISA kit for TNF-α, IL-1β and IL-6 (Thermo, USA) following the manufacturer’s instruction.

### mRNA sequencing

The macrophages were treated with and without C_2_S (150 µg/ml) for 24 h were used for RNA sequencing. Total RNA was extracted by using the Trizol kit (Invitrogen, Carlsbad, CA, USA), according to the manufacturer’s protocol. The RNA concentration was determined using Qubit, and the RNA amount and purity of each sample were assessed with a NanoDrop spectrophotometer (NanoDrop2000, Wilmington, DE, USA). RNA was isolated in 40 µl of DEPC water and stored at −80°C. RNA-seq libraries were prepared by using the Illumina TruseqTM RNA sample prep Kit and were sequenced using an Illumina NovaSeq PE150. Low-quality base and adapters were removed by using “fastp” [34]. Clean reads were aligned to the mouse genome (mm10) reference genome using STAR [35]. Transcript per million value and count number of reads mapped to each gene by using RNA-Seq by expectation-maximization [36]. And the ‘edgeR’ R software package was used to calculate the differential expression analysis between the two groups [37]. Kyoto Encyclopedia of Genes and Genomes (KEGG) analysis was performed using the ‘clusterProfiler’ R package [38]. KEGG terms with corrected *P* < 0.05 were considered significantly enriched.

### Analysis of mitochondrial function and autophagy in macrophages

#### Reactive oxygen species generation

The C_2_S extract-induced oxidative stress response in the macrophage was analyzed using a reactive oxygen species (ROS) kit (BestBio, China) as described previously [39]. RAW264.7 cells were cultured with C_2_S extract (150 μg/ml) for 4 h. ROS generation efficiency was visualized under a confocal microscope using a green laser (502 nm) (Leica, TCS SP8, Wetzlar, Germany). ROS quantitative analysis was performed in a fluorescence microplate reader with an excitation wavelength of 502 nm and an emission wavelength of 530 nm. Hydrogen peroxide (100 μM) was used as a ROS inducer positive control.

#### Mitochondrial membrane potential analysis

Mitochondrial membrane potential (MtMP) was analyzed using a JC-1 kit (BestBio, China) according to the manufacturer’s instructions. RAW264.7 cells were cultured with C_2_S extract (150 μg/ml) for 4 h. A confocal fluorescence microscope with the laser (488 nm) was used to visualize the fluorescence of the JC-1 monomers and aggregates. Flow cytometry analysis was used to quantify the MtMP of the macrophages. Carbonyl cyanide *m*-chlorophenylhydrazone (CCCP, 10 μM) was used as a positive control in all the mitochondrial function and autophagy-related experiments.

#### Intracellular calcium ion concentration analysis

The cytoplasmic free calcium ion concentration in macrophages upon treatment with C_2_S was analyzed by the intracellular calcium staining kit (Bestbio, China). RAW264.7 cells were cultured with C_2_S extract (150 μg/ml) for 4 h. A confocal fluorescence microscope with the laser (488 nm) was used to visualize the intracellular calcium. Flow cytometry (526 nm) analysis was used to quantify the concentration of free calcium ions in the cytoplasm.

#### Adenosine triphosphate production

ATP production in macrophages was analyzed using a bioluminescence ATP assay kit (Beyotime, China). Briefly, after culture with C_2_S extract (150 μg/ml) for 24 h, the RAW264.7 cells were lysed using the lysate solution provided in the kit, centrifuged at 12 000 g (4°C for 5 min), and the supernatant was collected. The intracellular ATP was analyzed according to the manufacturer’s instructions.

#### Autophagy analysis

Autophagy in macrophages was detected by enumerating the autophagosome formation using MDC (Monodansyl cadaverine) kit (Bestbio, China) and MitoTracker (Thermo, USA) following the manufacturer’s instruction by confocal microscopy and flow cytometric analysis. Briefly, the C_2_S extract (150 μg/ml) treated macrophages were cultured for 4 h and then stained with 50 μM of MDC and costained with 500 nM of MitoTracker and incubated in the dark for 20 min, After incubation, the cells were washed with 1× PBS and the autophagosome formation was visualized under confocal microscopy with a blue laser (335 nm) and the mitochondria were visualized with a red laser (579 nm). The autophagosome formation was quantitatively analyzed by using flow cytometry (518 nm).

### Transmission electron microscope analysis

TEM analysis was performed to observe the formation of autophagosomes and the morphology of mitochondria. Macrophages were cultured with or without C_2_S extract (150 μg/ml). After 4 h culture, cells were fixed with precooled glutaraldehyde and stored at 4°C overnight. The images were taken by using TEM (Tecnai Spirit, FEI, Hillsboro, USA) with maximum resolution and magnification as described previously [40].

### Western blot analysis

The cells were lysed with radioimmunoprecipitation lysis buffer (Beyotime, China) containing protease inhibitors (PMSF, Beyotime, China) on ice for 20 min. Cell lysates were used for western blot assay of Beclin1, LC3B and TOMM20. For PINK1 and Parkin, western blot analysis mitochondrial protein was extracted using a cell mitochondrial isolation kit (Beyotime, China). The protein concentrations in cell lysate and mitochondrial lysate were determined with a BCA protein assay kit (Bestbio, China). Lysate containing 20 μg protein was separated using 10–15% SDS-PAGE and then transferred to polyvinylidene difluoride (PVDF) membranes (Millipore, Billerica, MA, USA) using wet blotting techniques. The PVDF membranes were blocked with 3% skim milk powder for 1 h and washed thrice with 1× PBST and then incubated overnight at 4°C with primary antibodies. Protein from macrophages was detected using primary antibodies against PINK1 (Abcam, Cambridge, UK), Parkin (Abcam, Cambridge, UK), Beclin1 (Abcam, Cambridge, UK) and LC3B (Abcam, Cambridge, UK) in 1:2000 dilution. Protein from BMSCs was detected using primary antibody against RUNX2 (Abcam, Cambridge, UK), OPN (Bioss, USA) and GAPDH (Abcam, Cambridge, UK). After three washes with PBST, the PVDF membranes were further incubated with horseradish peroxidase (HRP)-conjugated secondary antibodies (Cell Signaling Technology, Beverly, MA, USA, 1:3000 dilution) for 2 h and the bands were visualized using suitable chemiluminescent substrate and the band images were captured by using Gel doc system equipped with a luminometer Gelview 6000 Pro (BLT, Guangzhou, Guangdong, China). GAPDH (Cell Signaling Technology) was used as a housekeeping protein for quantitative analysis of Beclin1, LC3B and TOMM20. β-tubulin (Cell Signaling Technology) was used as a housekeeping protein for quantitative analysis of PINK1 and Parkin. The Image J software (http://rsb.info.nih.gov/ij/) was used to quantify the band densities.

### Inhibition of autophagy in C_2_S-treated macrophages

An autophagy inhibitor 3-methyladenine (3MA, 0.1 mM) was added in macrophage culture to block autophagy. After 1 h, C_2_S (150 μg/ml) was added to the culture. After culturing for 24 h, the conditioned medium was collected and the concentrations of TNF-α, IL-1β and IL-6 were detected by ELISA.

### Osteogenic differentiation of bone marrow-derived mesenchymal stem cells

After 24 h of macrophage culture with or without C_2_S, the cultures were washed with PBS to eliminate C_2_S extract from the culture. The fresh culture medium was added to the culture and the conditioned medium (CM) was collected after 12 h of incubation. This CM contains C_2_S-free secretomes produced by the C_2_S-treated macrophages. Use of this macrophage-CM during BMSCs culture rules out the possible direct effect of C_2_S on osteogenic differentiation of BMSCs. Mouse BMSCs were cultured with CM and high glucose DMEM (in 1:1 ratio) in the presence of an osteogenic medium (OM) (10 nM dexamethasone, 50 μg/ml ascorbic acid, 10 mM β-sodium glycerophosphate). A total of three groups were allocated in this group: control group (OM), CM group (macrophage-CM and OM, 1:1 ratio) and C_2_S-CM group (C_2_S-treated macrophage-CM and OM, 1:1 ratio). All these three groups were maintained for osteogenic differentiation and the media were changed every 3–4 days time interval.

#### Alkaline phosphatase staining and activity

ALP staining and ALP activity were performed in osteogenic induced BMSCs with or without C_2_S at day 10. ALP staining was performed with the BCIP/NBT alkaline phosphatase color kit (Nanjing Jiancheng Biology Engineering Institute, China) according to the manufacturer’s instructions. ALP activity was performed using an ALP detection kit (Bestbio, China) as described previously [7]. Total protein concentration in the cell lysate was analyzed by the BCA protein assay kit. The ALP activity was normalized to the total protein content.

#### Alizarin red staining

Alizarin red (Sigma, USA) staining was performed on day 14 to visualize the mineralized matrix in BMSCs culture. For semiquantitative analysis of 200 μl of 10% CPC (Sigma, USA) solution was added to each well (48-well culture plate) to dissolve the mineralized nodules. The supernatant was transferred to a 96-well plate, optical density was measured at 562 nm, quantitative analysis was performed as described previously [7].

#### RT-qPCR analysis

The total RNA was extracted from BMSCs culture on day 7 using RNAEX reagent (Accurate Biotechnology, Hunan, China). Extracted 500 ng total RNA from each sample was reverse transcribed to cDNA using Evo M-MLV RT Premix for qRT-PCR (Accurate Biotechnology, Hunan, China). The reverse-transcribed cDNA was subjected to RT-qPCR (SYBR Green Premix Pro Taq HS qPCR Kit, Accurate Biotechnology, Hunan, China) using the following cycling conditions: 95°C for 10 min (initial denaturation), 40 cycles of 95°C for 15 s and 60°C for 60 s and then 60°C for 5 min for the terminal extension. The temperature was increased by 1 degree every 20 s to obtain the melting curve. The relative quantitative ^ΔΔ^Ct method was used to determine the fold change of expression. The expressions of osteogenic gene RUNX2, ALP, OCN and BMP2 were analyzed by RT-qPCR. GAPDH was used as an internal control to normalize the expression of specific genes. The primer sequences used for RT-qPCR are listed in Table 1.

**Table 1.** Primers used for RT-qPCR

Gene	Forward primer (5′-3′)	Reverse primer (5′-3′)
GAPDH	TGTGTCCGTCGTGGATCTG	TTGCTGTTGAAGTCGCAGGA
RUNX2	CACTGGCGGTGCAACAAGA	TTTCATAACAGCGGAGGCATTTC
ALP	TGCCTACTTGTGTGGCGTGAA	TCACCCGAGTGGTAGTCACAATG
OCN	GCTTGTGACGAGCTATCAGACCAG	AGCTGCTGTGACATCCATACTTGC
BMP2	GAATGACTGGATCGTGGCACCTC	GGCATGGTTAGTGGAGTTCAGGTG

### Statistical analysis

The data was analyzed using SPSS 20.0 software (SPSS, Inc., Chicago, Illinois). All data are expressed as mean ± SD. All experiments were performed at least three times. Differences between groups were analyzed using analysis of variance followed by Bonferroni *post hoc* comparison and Dunnett’s test, when appropriate. When *P* values < 0.05, it is considered statistically significant.

### Availability of data and materials

All raw sequencing data can be accessed from the Sequence Read Archive (project accession GSE171979).

## Results

### Characteristics of C_2_S particles

Based on the results from the scanning electron microscopy, the C_2_S particles appear as spherical particles aggregated in blocks (Supplementary Fig. S1). The laser particle size analysis showed that the main size of the particles is about 9.41±0.65 μm. The energy dispersive spectrometry showed that the surface of the particles is mainly calcium, silicon, oxygen and carbon. X-ray diffraction analysis showed that the particle collection of illustration matched calcium silicate-matching Ca_2_SiO_4_. Inductively coupled plasma optical emission spectrometer showed that C_2_S particles dissolved in DMEM release calcium, silicon and phosphorus ions.

### Effect of C_2_S on macrophage viability, inflammation induction and oxidative stress response

**Figure 1.**
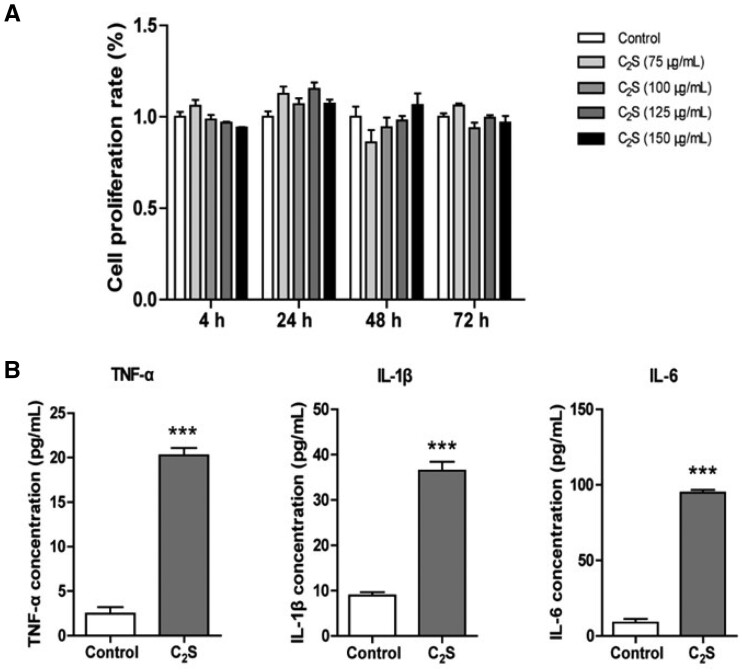
C_2_S extract did not affect macrophage viability but induced macrophagic inflammation. (**A**) Macrophage viability, and (**B**) TNF-α, IL-1β and IL-6 expression pattern in macrophage analyzed by ELISA. Data are presented as mean±SD, *n* = 3. Significant effect of the treatment compared with the control group, ***P < 0.001

**Figure 2.**
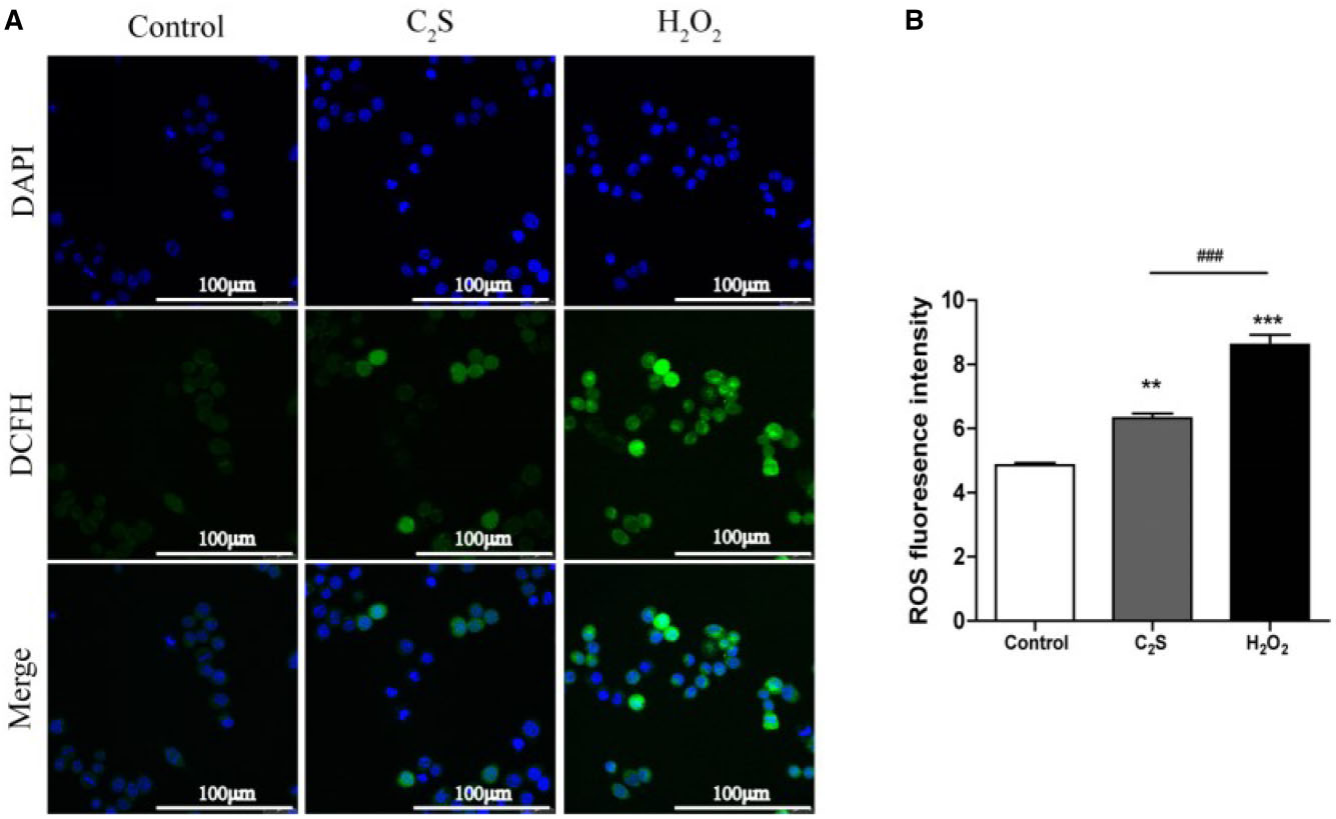
C_2_S-induced oxidative stress in macrophages. (**A**) Representative fluorescence microscopy images showing ROS-stained macrophages. (**B**) Quantification of ROS fluorescence intensity by flow cytometry analysis. Data are presented as mean±SD, *n* = 3. Significant effect of the treatment: compared with the control group, **P < 0.01, ***P < 0.001; compared with the positive control group (H_2_O_2_), ^###^*P* < 0.001

Different concentration C_2_S extracts did not affect macrophage viability, ruling out any cytotoxic effect of C_2_S on macrophages (Fig. 1A). Results from our previous studies showed that C_2_S triggers macrophagic inflammation [2, 4, 10]. Based on the result of cell viability, we tested the inflammation induction potential of the highest concentration of C_2_S (150 μg/ml) extract tested in this study on inflammatory cytokine release by macrophages. C_2_S extract treatment for 24 h robustly enhanced TNF-α, IL-1β and IL-6 production by macrophages (Fig. 1B). The C_2_S extract induced ROS production in macrophages by 1.3-fold compared with control (Fig. 2A and B).

### mRNA sequencing data analysis

**Figure 3.**
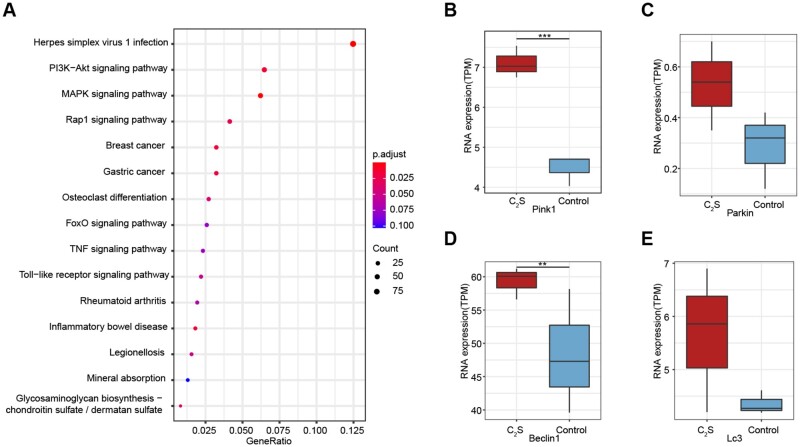
C_2_S extract treatment regulated the expression of mitochondrial function/autophagy-related genes and signaling pathways. (**A**) KEGG pathway enrichment analysis of differentially expressed genes. (**B**–**E**) The expression of PINK1, Parkin, Beclin1 and LC3 was analyzed by RNA-sequencing

Results of mRNA sequencing showed that the C_2_S-treated macrophages showed differential mRNA expression patterns compared with the control group. A total of 1475 mRNAs were differentially upregulated and 924 mRNAs were differentially downregulated in C_2_S-treated macrophages (Supplementary Figs S2 and S3). The R package ‘ClusterProfiler’ was used to perform KEGG pathway enrichment analysis of these differentially expressed mRNAs. We found that differentially expressed mRNAs are mainly distributed in the essential signaling pathways involved in mitochondrial function and autophagy, for example MAPK, PI3K-Akt, TNF and Rap1 signaling pathway (Fig. 3A). Among the mitochondrial function and autophagy-related mRNAs, PINK1, Parkin, Beclin1 and LC3 were differentially upregulated (Fig. 3B–F). Based on these results, we choose to analyze mitochondrial function and autophagy as a possible mechanism of C_2_S-induced macrophagic inflammation.

### Effect of C_2_S on macrophage mitochondrial function

**Figure 4.**
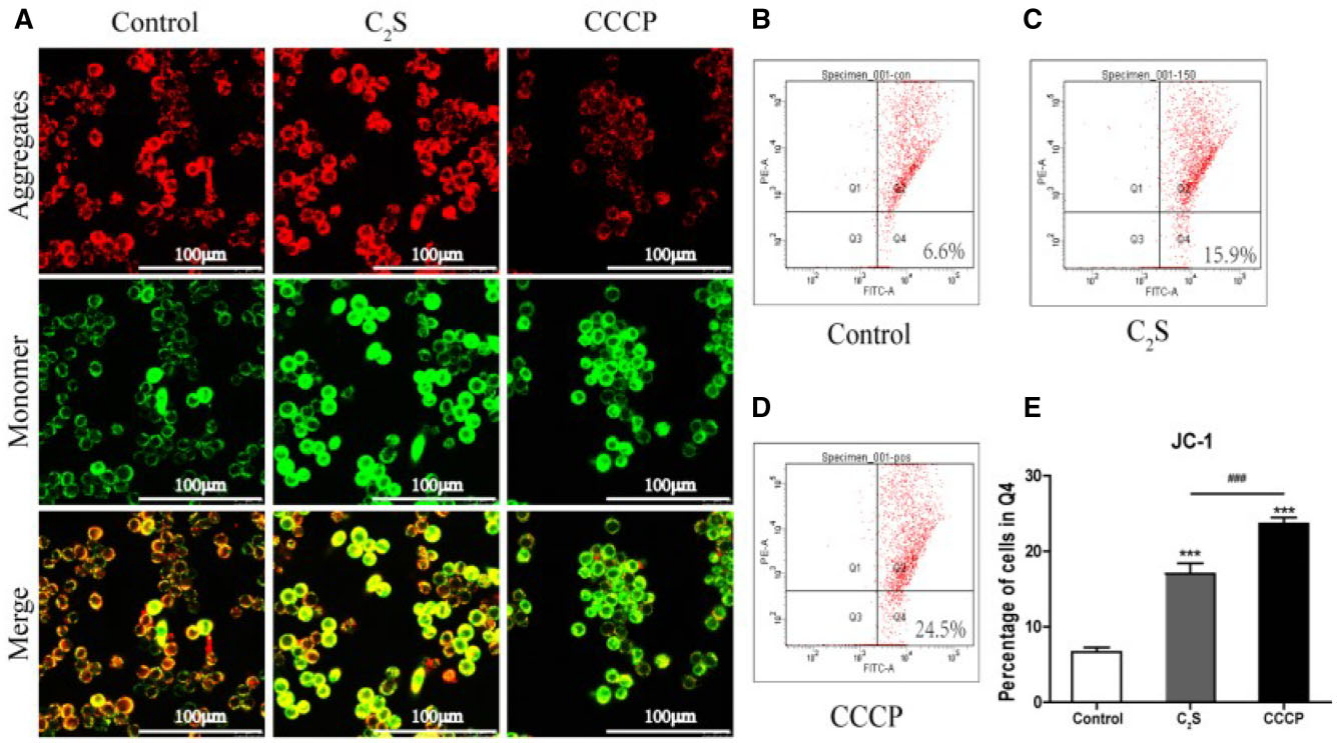
C_2_S reduced macrophagic MtMP. (**A**) Representative fluorescence microscopy images showing JC-1-stained macrophages. (**B**–**D**) Representative flow cytometry analysis histogram images showing JC-1-stained cells in the Q4 region. (**E**) Flow cytometry quantitative analysis of the percentage of cells in Q4 region. Data are presented as mean±SD, *n* = 3. Significant effect of the treatment: compared with the control group, ***P < 0.001, compared with the positive control group (CCCP), ^###^*P* < 0.001

MtMP reflects electron transport and oxidative phosphorylation process and is an indicator of mitochondrial activity. MtMP was analyzed using JC-1 dye. This dye exhibits MtMP-dependent accumulation in mitochondria, indicated by a fluorescence emission shift from red to green when MtMP declines due to membrane damage (Fig. 4A). The C_2_S extract-treated macrophages showed higher intensity of JC-1 staining (green fluorescence) compared with the control group (Fig. 4A). The quantitative analysis of JC-1 stained cells showed 2.55-fold higher numbers of JC-1 stained macrophages in the C_2_S-treated group compared with the control group (Fig. 4B). This result indicates that the C_2_S impairs the mitochondrial activity of macrophages.

**Figure 5.**
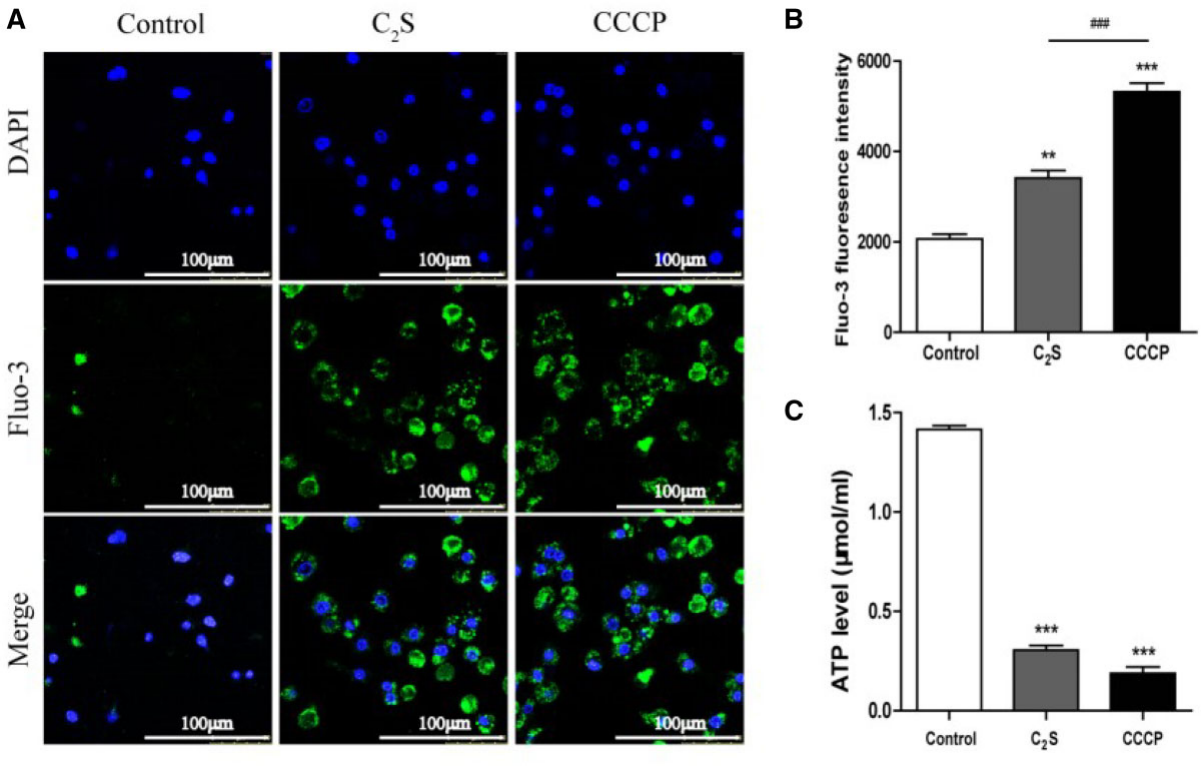
C_2_S enhanced intracellular calcium levels and reduced ATP production in macrophages. (**A**) Representative fluorescence microscopy images of Flu-3 stained, macrophages. (**B**) Quantitative analysis of Fluo-3-stained macrophages by flow cytometry analysis. (**C**) Quantitative measurements of ATP level in macrophages. Data are presented as mean±SD, *n* = 3. Significant effect of the treatment: compared with the control group, **P < 0.01, ***P < 0.001, compared with the positive control group (CCCP), ^###^*P* < 0.001

The disrupted mitochondrial function increases intracellular calcium ion concentration. We analyzed the intracellular calcium ion concentration in C_2_S extract-treated macrophages using the Fluo-3 probe. C_2_S extract-treated macrophages showed higher intensity of green color Fluo-3 compared with the control group (Fig. 5A). Quantitative analysis of Fluo-3 staining intensity in macrophages by flow cytometry analysis revealed a 2.33-fold overload of calcium ion in the C_2_S extract-treated group compared with the control group (Fig. 5B). The ATP production decreases during mitochondrial dysfunction [41]. The ATP level was drastically reduced in C_2_S extract-treated macrophages compared with the control group (Fig. 5C). The results of calcium ion concentration and ATP release assays indicate the disrupted mitochondrial function in C_2_S extract-treated macrophages.

### C_2_S affects mitochondrial morphology and induces autophagy

**Figure 6.**
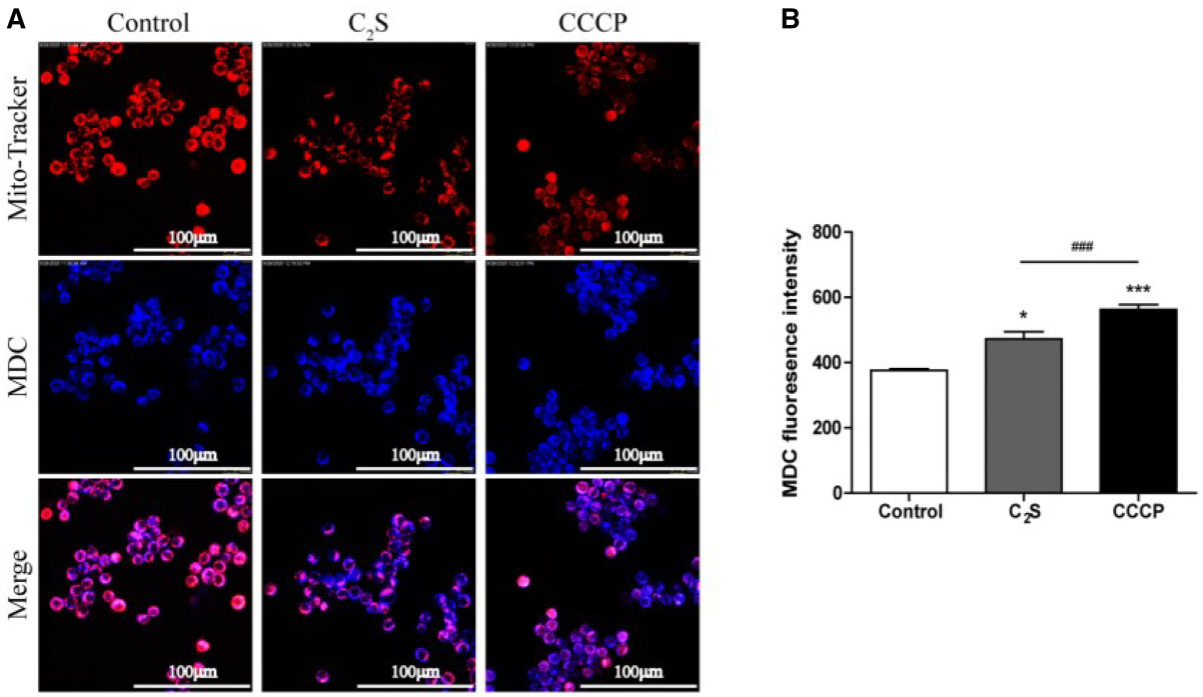
C_2_S-induced autophagy in macrophages. (**A**) Representative fluorescence microscopy images showing mitochondria and autophagosomes in macrophages. (**B**) Quantitative analysis of MDC-stained fluorescence intensity by flow cytometry analysis. Data are presented as mean±SD, *n* = 3. Significant effect of the treatment compared with the control group: *P < 0.05, ***P < 0.001, compared with the positive control group (CCCP), ^###^*P* < 0.001

**Figure 7.**
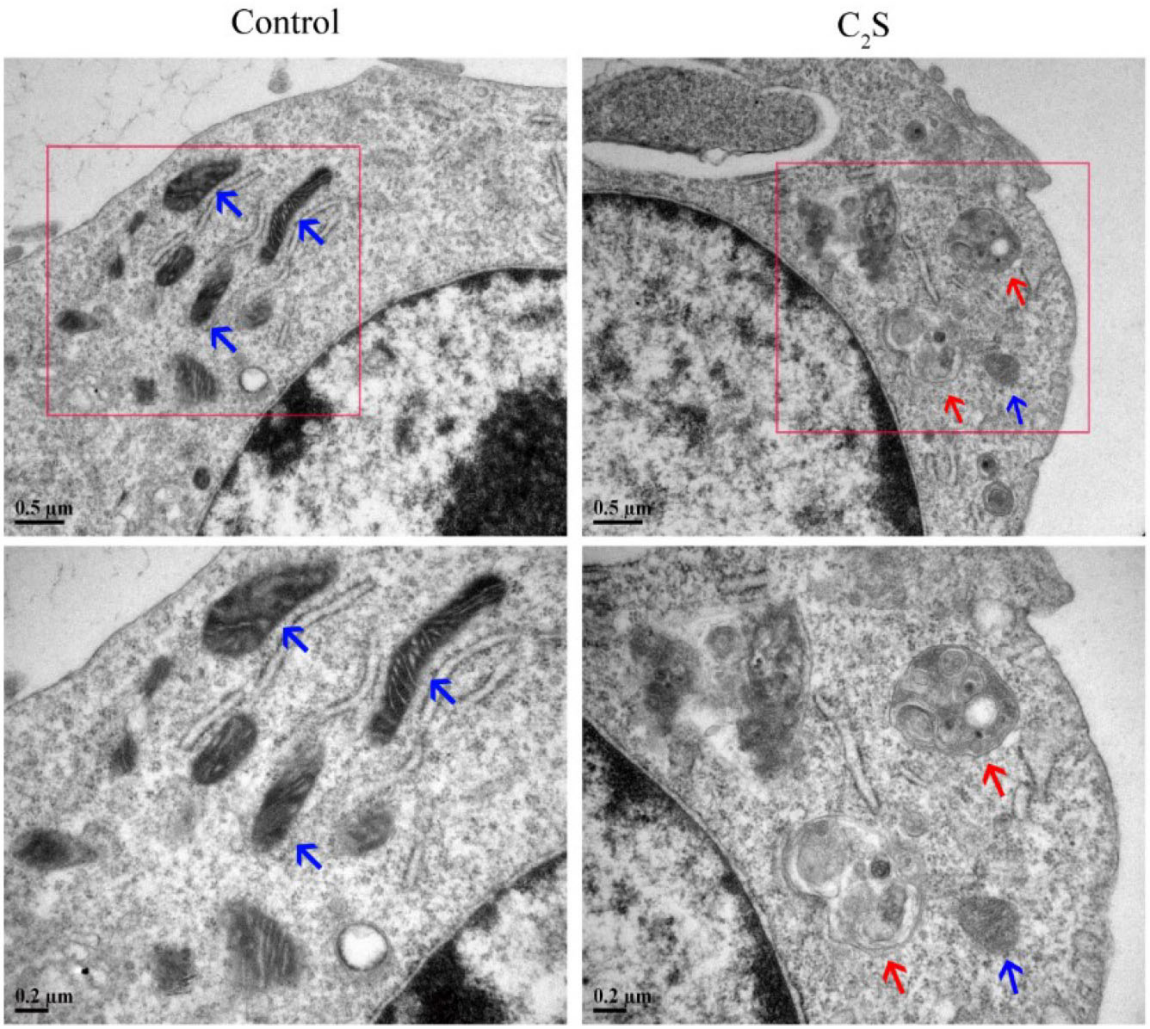
C_2_S altered mitochondrial morphology changes and autophagosome formation in macrophages. The mitochondria and autophagosomes were observed under a transmission electron microscope. The blue arrows point to mitochondria and the red arrows point to autophagosomes

Mitochondrial dysfunction induces autophagy and macrophagic inflammation [17]. MDC/MitoTracker staining showed more numbers of autophagosomes around the mitochondria and a reduced number of healthy mitochondria in the C_2_S-treated macrophages compared with the control group (Fig. 6A). Flow cytometry analysis revealed the 1.26-fold higher intensity of blue MDC staining in C_2_S-treated macrophages compared with the control group (Fig. 6B). TEM images showed abundant mitochondria with normal morphology in the control group (Fig. 7). In contrast, swollen mitochondria surrounded by autophagosomes were visualized in the C_2_S-treated macrophage (Fig. 7).

### Expression pattern of mitochondrial function and autophagy-related proteins

**Figure 8.**
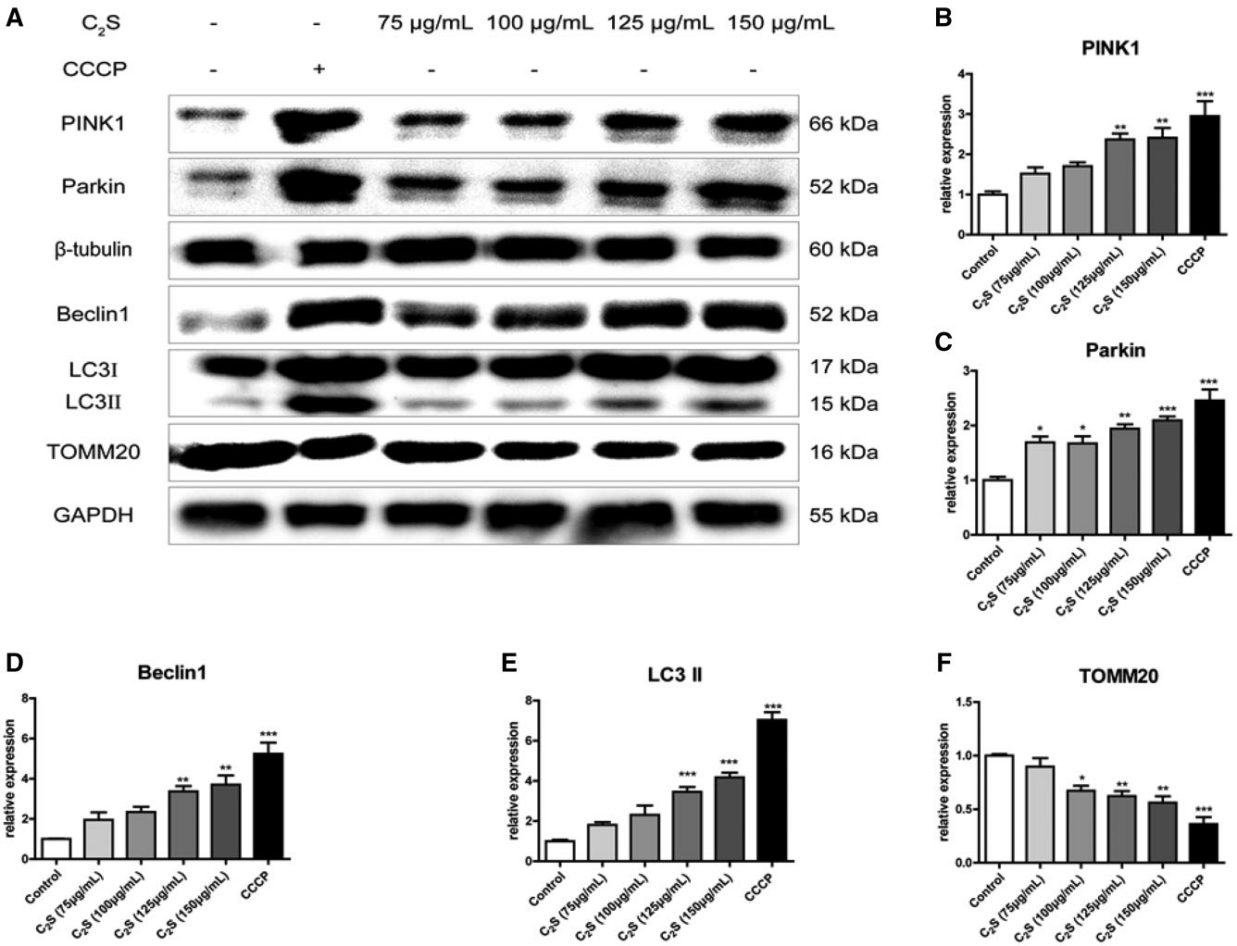
C_2_S treatment for 24 h promoted the expression of autophagy-related proteins in macrophages and downregulated the outer mitochondrial membrane protein TOMM20. (**A**) Western blots showing expression of mitochondrial function and autophagy-related proteins. (**B**–**F**) Quantification of western blot: PINK1, Parkin, Beclin1, LC3 and TOMM20. Significant effect of the treatment: *P < 0.05, **P < 0.01 and ***P < 0.001

Western blot images and quantitative analysis showed that the C_2_S extract dose dependently enhances PINK1, Parkin, Beclin1 and LC3 expression in macrophages (Fig. 8A). The expression of TOMM20 has reduced in C_2_S extract-treated macrophages in a dose-dependent manner (Fig. 8A). C_2_S extract (150 μg/ml) treatment upregulated PINK1, Parkin, Beclin1 and LC3 expression in macrophages by 2.41-, 2.09-, 3.7- and 4.18-fold, respectively, compared with control (Fig. 8B–E). C_2_S extract (150 μg/ml) treatment downregulated TOMM20 expression in macrophages by 0.56-fold compared with control (Fig. 8F). These results further proved that C_2_S played a major role in C_2_S on mitochondrial dysfunction and autophagy-mediated macrophagic inflammation induction.

### Partial inhibition of autophagy reversed the C_2_S-induced inflammatory response

**Figure 9.**
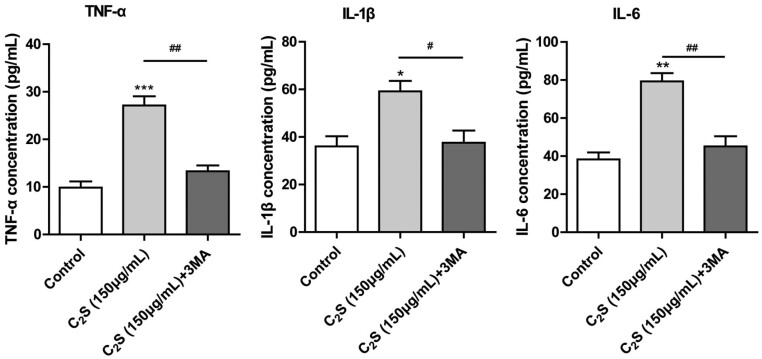
The expression level of TNF-α, IL-1β and IL-6 in the cell culture supernatant was analyzed by ELISA. Significant effect of the treatment compared with the control group: *P < 0.05, **P < 0.01, ***P < 0.001, ***P < 0.001, compared with the C_2_S group, ^#^*P* < 0.05, ^##^*P* < 0.01

To further analyze the role of autophagy on C_2_S-induced macrophagic inflammation, autophagy was partially inhibited in macrophage culture using 0.1 mM 3MA. Inhibition of autophagy in macrophages almost reversed the C_2_S-induced overexpression of inflammatory markers TNF-α, IL-1β and IL-6 (Fig. 9).

### C_2_S-treated macrophages promote osteogenic differentiation of BMSCs

**Figure 10.**
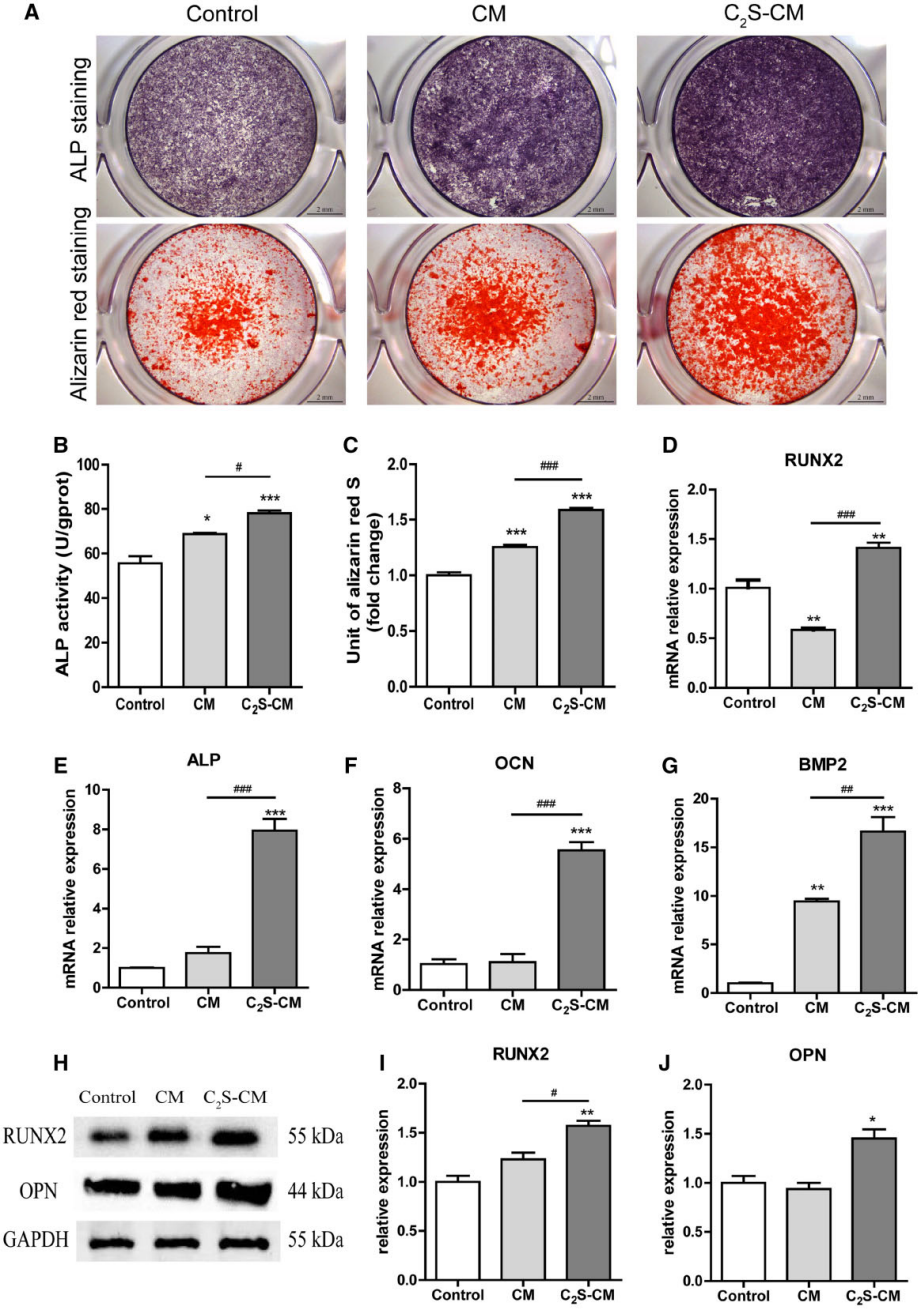
C_2_S-treated macrophages enhanced the osteogenic differentiation of BMSCs and enhanced the expression of osteogenic markers. (**A**) ALP staining and Alizarin red staining. (**B**) ALP activity. (**C**) Quantification of alizarin red staining. (**D**–**G**) mRNA expression pattern of osteogenic markers at day 7. (**H**–**J**) Protein expression pattern of osteogenic markers at day 14. Significant effect of the C_2_S group compared with the control group: *P < 0.05, **P < 0.01 and ***P < 0.001, and compared with the CM group: ^#^*P* < 0.05, ^##^*P* < 0.01 and ^###^*P* < 0.001. Control: BMSCs cultured in OM, CM: BMSCs cultured in OM with macrophage-CM and C_2_S-CM: BMSCs cultured in OM with C_2_S-treated macrophage-CM

Osteogenesis is the key phenomenon of bone defect healing. Since C_2_S induces macrophagic inflammation via mitochondrial dysfunction and autophagy, we further tested the effect of C_2_S-induced macrophagic inflammation on the osteogenic differentiation of precursor cells. C_2_S-treated-macrophage-CM induced higher ALP production in BMSCs compared with macrophage-CM or control group (Fig. 10A). ALP activity analysis revealed that C_2_S-treated macrophage-CM enhanced ALP activity in BMSCs by 1.4-, and 1.14-fold compared with macrophage-CM and control group, respectively (Fig. 10B). Alizarin red staining images showed higher intensity of mineralized matrix in C_2_S-treated macrophage-CM group compared with control or macrophage-CM group (Fig. 10A). Quantitative analysis of alizarin red staining showed 1.59-, and 1.26-fold higher mineralized matrix in C_2_S-treated macrophage-CM group compared with macrophage-CM and control group, respectively (Fig. 10C). Similarly, C_2_S-treated macrophage-CM upregulated mRNA expression of osteogenic markers Runx2, ALP, OCN and BMP2 (Fig. 10D–G). The C_2_S-treated macrophage-CM also induced the protein level expression of osteogenic markers RUNX2 and OPN in BMSCs (Fig. 10H–J). These results indicate the anabolic effect of C_2_S-induced macrophagic inflammation on osteogenic differentiation of precursor cells.

## Discussion

**Figure 11.**
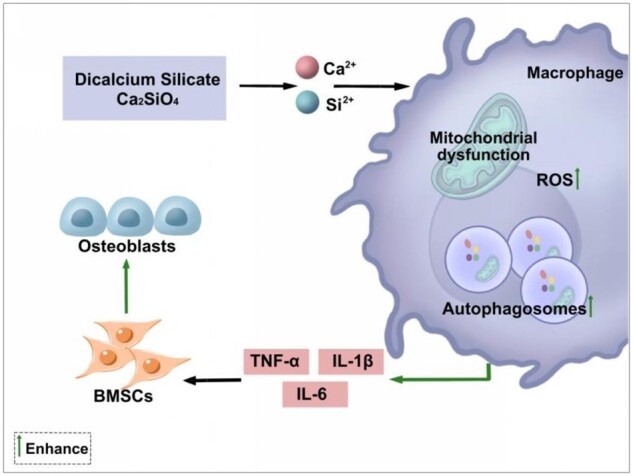
C_2_S-induces macrophagic inflammation via mitochondrial dysfunction and autophagy that promotes osteogenic differentiation of precursor cells

C_2_S releases calcium and silica ions that affect the function of cells involved in bone regeneration [2, 3, 10, 42]. Results of previous studies from our research group and reports from literature had unraveled the anabolic effect of C_2_S on osteogenic differentiation of precursor cells and the underlying molecular mechanisms [4, 7, 43]. Macrophages played a vital role in immunoregulation during bone defect healing [8, 44, 45]. The development of macrophage-mediated immunomodulatory biomaterials is currently a hot research topic of bone tissue engineering. Results from our previous studies showed that C_2_S regulates macrophagic inflammation [2, 4, 10]. In this study, we unraveled that the C_2_S induces macrophagic inflammation via mitochondrial dysfunction and autophagy. Moreover, the C_2_S-induced macrophagic inflammation robustly promoted osteogenic differentiation of BMSCs (Fig. 11). Our results showed that the C_2_S-induced macrophage-mediated immunomodulation positively regulates osteogenesis, indicating the potential application of C_2_S in developing immunomodulatory biomaterials for bone tissue engineering and implantology applications.

Reports from literature show that various immunomodulatory biomaterials have been designed to affect macrophage proliferation and activation [27, 46–49]. In this study, C_2_S extracts (75, 100, 125, or 150 μg/ml) did not affect macrophage viability. This finding is in accordance with the findings of previous studies [2, 10]. Our previous study investigated the effect of C_2_S extract up to the dose of 100 μg/ml on macrophage cell viability, inflammatory markers production and ROS production [2]. In the current study, we observed no cytotoxic effect of 150 μg/ml of C_2_S extract on macrophages. Therefore, we chose the highest concentration tested (150 μg/ml) to further analyze the effect of C_2_S on macrophagic inflammation. Moreover, among the concentration tested (75, 100, 125, 125 and 150 μg/ml), the 150 μg/ml of C_2_S extract showed the highest effect on autophagy and mitochondrial function markers expression in macrophages, indicating 150 μg/ml as a suitable concentration for the scope of this study (Fig. 8). We found that C_2_S treatment promoted the expression of proinflammatory cytokines TNF-α, IL-1β and IL-6 in macrophages. Our previous studies had also reported a similar effect of C_2_S on macrophages [2, 4, 10, 42]. Results from this study and reports from literature elucidate the macrophagic inflammation-inducing potential of C_2_S [4, 9, 10, 42]. However, the mitochondrial function of C_2_S-treated macrophages is still not fully understood. In this study, we attempt to investigate the mitochondrial function and autophagy-mediated effect of C_2_S on the induction of macrophagic inflammation.

The ionic products released by silicon-based biomaterials affect mitochondrial membrane functions [50]. Mitochondrial dysfunction induces macrophagic inflammation [16]. We analyzed the effect of C_2_S on the mitochondrial function of macrophages. The C_2_S treatment robustly upregulated ROS production and intracellular calcium overload and reduced MtMP and ATP production in macrophages. TOMM20 plays a key role in mitochondrial protein import [51]. C_2_S-treated macrophages showed downregulation of TOMM20 expression. C_2_S treatment also altered the mitochondrial morphology in macrophages. These results indicate that the C_2_S-induced mitochondrial dysfunction in macrophages.

C_2_S causes oxidative stress and mitochondrial damage in RAW264.7 cells, but it does not significantly inhibit the growth and proliferation of RAW264.7 cells. Autophagy prevents cell apoptosis and inflammation by forming autophagosomes in cells to remove the excessive accumulation of ROS in cells and removing damaged mitochondria [52]. Based on the aforementioned facts, we speculated that autophagy may be a ‘self-rescue’ mechanism in cells that can resist the increase of ROS and the change of mitochondrial outer membrane potential. Autophagy plays important role in macrophage activation [53], via various signaling pathways, including, NF-κB, MAPK, TNFα, PI3K/Akt and mTOR [54, 55]. The damaged mitochondria upregulate PINK1/Parkin expression [56]. Upregulated PINK1/Parkin is associated with macrophagic inflammation [57]. C_2_S treatment robustly upregulated mitochondrial PINK1/Parkin expression in macrophages. Mitochondrial dysfunction induces autophagy in a tissue-specific manner [58]. Moreover, autophagy is involved in macrophage function and inflammatory responses [55]. In this study, C_2_S treatment-induced autophagy in macrophages as indicated by the higher numbers of autophagosomes in TEM images and MDC staining. The expression of autophagy-related proteins Beclin1 and LC3 II were robustly upregulated in C_2_S-treated macrophages. Enhanced LC3 II expression is associated with autophagy induction in various cells [59]. KEGG analysis of mRNA sequencing of C_2_S-treated macrophages revealed that the differentially expressed mRNAs were mainly distributed in the essential signaling pathways involved in mitochondrial function and autophagy, for example MAPK, TNF and PI3K-Akt signaling pathway. Findings from the literature and current study indicate the possible role of C_2_S-induced mitochondrial dysfunction and autophagy on the induction of macrophagic inflammation.

In the transient inflammatory response at the initial stage of bone repair and healing, the inflammatory macrophages (M1 phenotype) initially gathered at the repair site, which polarized into the M2 phenotype after about 3–4 days [27], and the whole process lasted about 7 days [60]. Early-stage inflammation is essential for the effective healing of bone defects [11, 13]. Macrophage polarization induced by biomaterials and implants regulates osteoinductivity and osseointegration [61]. The C_2_S-induced macrophagic inflammation robustly promoted the osteogenic differentiation of BMSCs. This result indicates that the C_2_S-induced macrophagic inflammation acts as an early-stage inflammation required for triggering bone healing. However, chronic and intense inflammation impairs bone defect healing. Macrophages with M2 phenotype after the initial stage promotes bone formation [62], and participate in the formation of new bone by secreting bone formation-related molecules such as IL-10, TGF-β and BMP2 [63]. In this study, we analyzed only the effect of 24 h C_2_S treatment on the induction of macrophagic inflammation. Our results indicated that the anabolic effect of C_2_S-induced early-stage macrophagic inflammation on osteogenesis. The switching of macrophage polarization from M1 and M2 after the early stage of healing is closely related to effective bone regeneration [11, 64]. Therefore, the long-term effect of C_2_S on macrophage activation and phenotype switching should be further analyzed to better understand the macrophage-mediated role of C_2_S during the whole bone regeneration process.

In this study, we extensively analyzed the mitochondrial function and autophagy in C_2_S-treated macrophages. Our results revealed that C_2_S-induced macrophagic inflammation, mitochondrial dysfunction and autophagy. C_2_S-induced macrophagic inflammation robustly promoted osteogenic differentiation of BMSCs. The limitation of this study is that we did not investigate the exact molecular mechanism of C_2_S-induced mitochondrial dysfunction and autophagy. Furthermore, *in vivo* bone defect healing effect of C_2_S-induced macrophagic inflammation should be further tested.

## Conclusions

C_2_S induces macrophagic inflammation, mitochondrial dysfunction and autophagy. Recent studies had reported the role of mitochondrial dysfunction and autophagy on macrophagic inflammation (M1 phenotype). Based on the findings from the literature and our current study, we speculate that the C_2_S regulates macrophagic inflammation possibly via inducing mitochondrial dysfunction and autophagy. The C_2_S-induced macrophagic inflammation robustly promoted osteogenic differentiation of BMSCs. Our results indicate the potential application of C_2_S in the fabrication of immunomodulatory biomaterials/implants with osteoinductive properties.

## Supplementary data

Supplementary data are available at *REGBIO* online.

## Funding

This study was supported by High-level University Construction Funding of Guangzhou Medical University (02-412-B205002-1003017 and 06-410-2106035).

## Author contributions

Q.Z., J.L.P., Q.L., X.L. and W.Z. designed the study, interpreted the data and revised the manuscript. Q.L., X.L. and W.Z. performed experiments, analyzed the data and prepared the first draft of the manuscript. W.C., M.Z., A.W., W.C., Z.Y., Q.H. and D.N. performed the experiments, performed the statistical analysis and prepared the figures. All authors approved the final version of the manuscript.

*Conflict of interest statement*. None declared.

## Supplementary Material

rbab075_Supplementary_Data
