# Supplementary material for: Dicalcium silicate-induced mitochondrial dysfunction and autophagy-mediated macrophagic inflammation promotes osteogenic differentiation of BMSCs
Source: Regen Biomater. 2021 Dec 13;9:rbab075. doi: 10.1093/rb/rbab075 (PMC9039510; doi:10.1093/rb/rbab075)
Supplement: rbab075_Supplementary_Data [file rbab075_supplementary_data.docx]

**Supplemetary file**

**
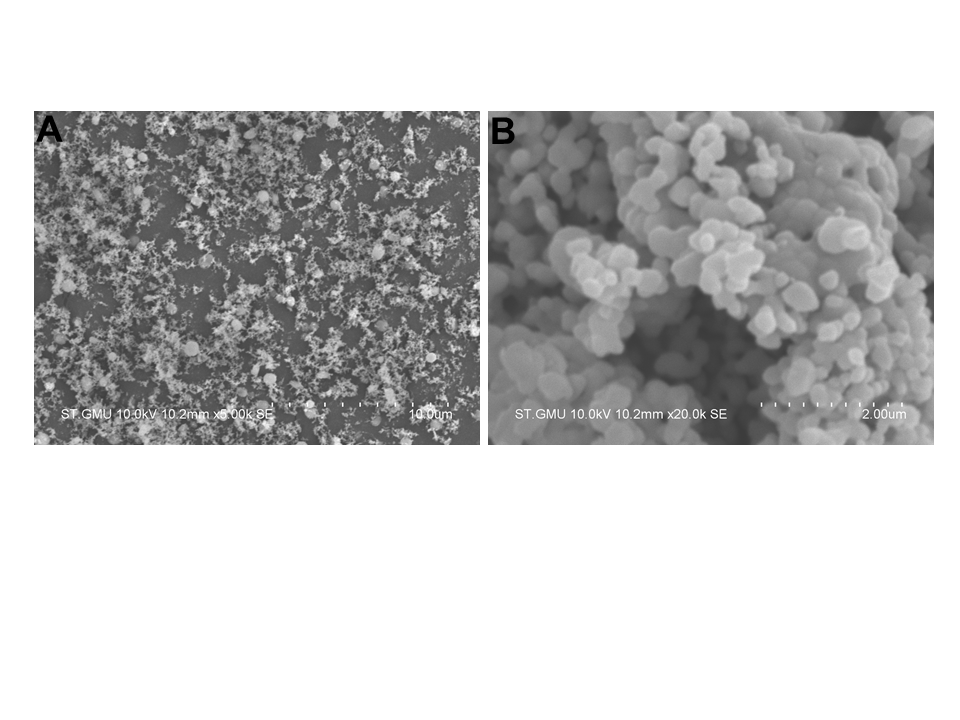
**

**Supplementary Figure 1.** SEM images of C_2_S particles at (A) low, and (B) high magnification.


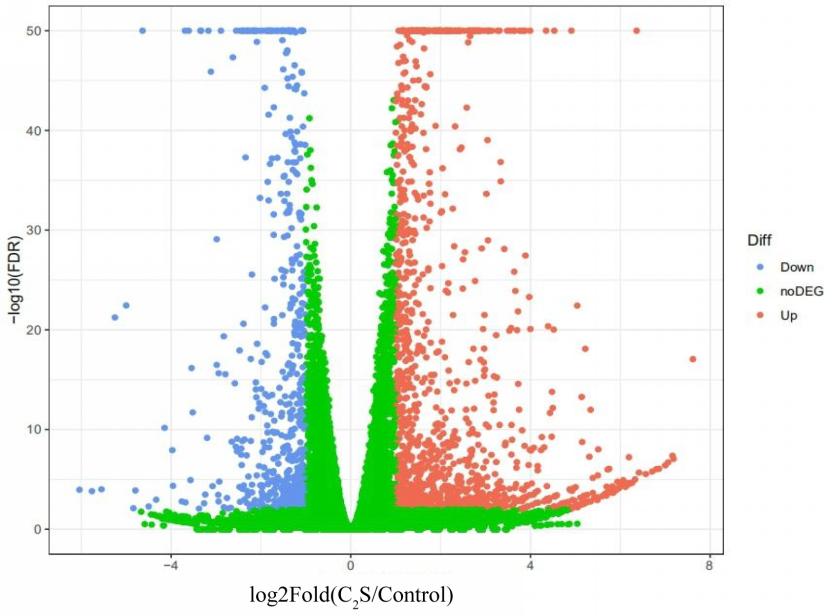


**Supplementary Figure 2.** Volcano plot shows the differentially expressed mRNAs in C_2_S-treated macrophages.


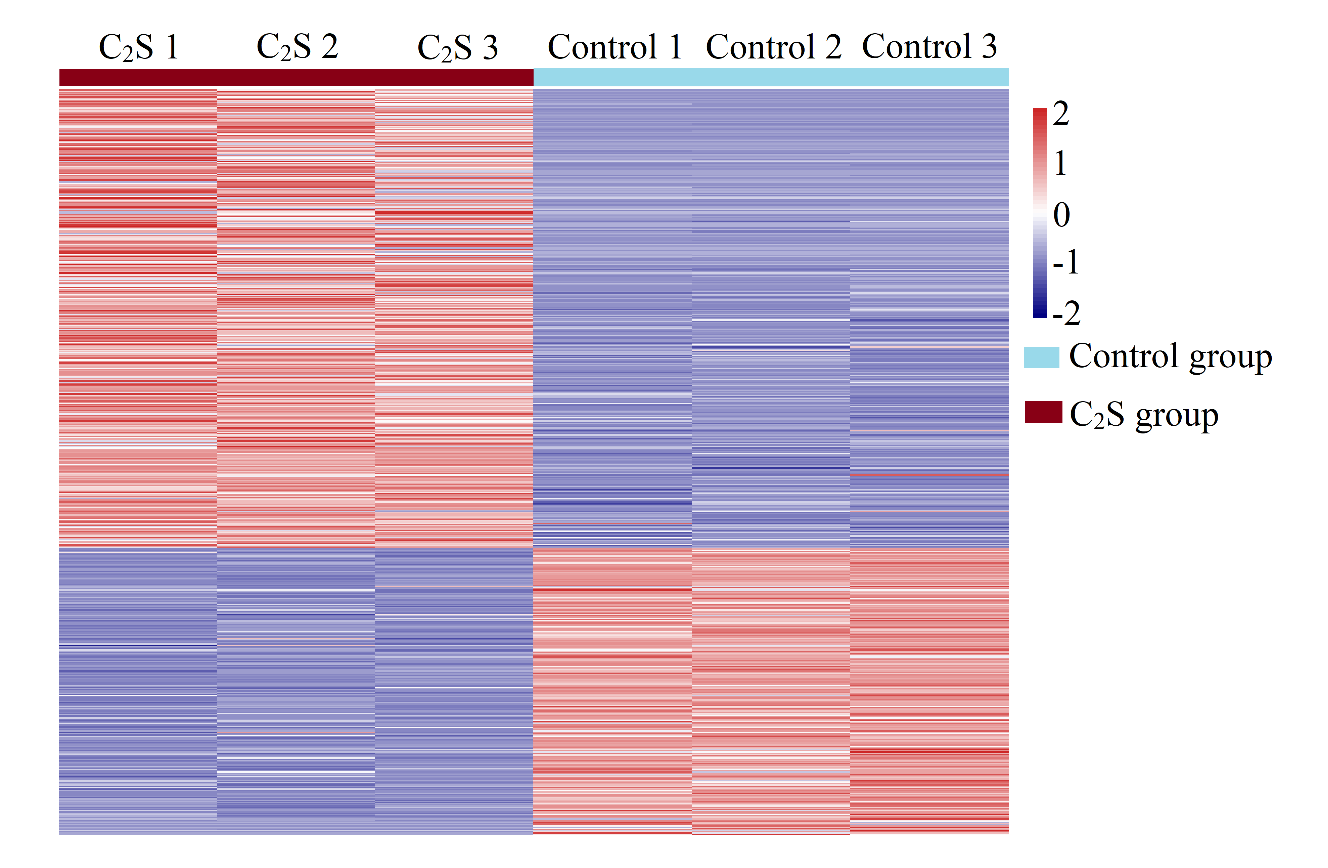


**Supplementary Figure 3.** Heat map shows the differentially expressed mRNAs in C_2_S-treated macrophages.
